# Advances in Alphavirus and Flavivirus Research

**DOI:** 10.3390/v16060882

**Published:** 2024-05-30

**Authors:** Young Chan Kim, Arturo Reyes-Sandoval

**Affiliations:** 1 Oxford Vaccine Group, Department of Paediatrics, University of Oxford, Oxford OX3 7LE, UK; 2 Division of Structural Biology, Wellcome Centre for Human Genetics, University of Oxford, Oxford OX1 2JD, UK; 3 Instituto Politécnico Nacional (IPN), Av. Luis Enrique Erro s/n. Unidad Adolfo López Mateos, México City 07738, Mexico; arturoreyess@ipn.mx

Newly emerging viruses, primarily zoonotic or vector-borne, pose a persistent threat to public health and have led to outbreaks of global concern. The recent COVID-19 pandemic has had a devastating impact on the world’s healthcare system and the global economy [1]. Flaviviruses and alphaviruses are single-stranded RNA viruses and are transmitted by *Aedes* mosquitoes, and they have the ability to unexpectedly (re)-emerge and cause severe viral infections in humans [2,3,4,5,6,7,8]. These flaviviruses and alphaviruses belong to the broader family of arboviruses. Over the past century, they have caused considerable disease loads and public health issues due to their global spread and transmission [6,7,8,9,10]. The present Special Issue focuses on disseminating the latest advancements in research on alphaviruses and flaviviruses. The articles featured in this Special Issue are categorised into three different topics related to the study of these viruses. 

The first section consists of 11 research articles contributed by different research groups. Nissly et al. investigated the susceptibility of chickens to experimental Zika (ZIKV) infection to show that chickens are not susceptible to ZIKV infection [11]. Through their study, they showed that ZIKV did not cause clinical signs in chickens of all age groups tested, and there was rapid clearance of ZIKV in older chickens, which coincided with an effective innate immune response, highlighting age-dependent susceptibility. 

Kizu et al. performed ELISA and neutralisation assays to show that there is potential serological misdiagnosis of Barmah Forest virus (BFV) and Ross River virus (RRV) as Chikungunya virus (CHIKV) infections in Australia [12]. This particular study highlights the need for diagnostic laboratory tests capable of distinguishing between CHIKV infections and actively co-circulating RRV and BFV. 

Souza et al. sequenced DENV-1 samples from 2015 to 2022 in Araraquara, Brazil, and studied the evolutionary history of DENV-1 in Araraquara using Brazilian and worldwide DENV-1 sequences [13]. They showed through their study that there have been at least three introductions of genotype V in Araraquara, distributed in two main lineages (L Ia and L Ib and L II), within the last ten years. 

Rainey et al. used the reporters for Semliki Forest virus (SFV) in *Aedes* albopictus cells to show that Wolbachia strains wMel, wAu, and wAlbB blocked viral replication and translation during the early stages of infection and that the storage of cholesterol in lipid droplets is not key to this inhibition [14]. Furthermore, these Wolbachia strains showed strong antiviral activity against another alphavirus, o’nyong nyong virus (ONNV), suggesting that there may be differences in how alphaviruses are inhibited by Wolbachia in comparison to flaviviruses. 

Michie et al. conducted whole-genome sequencing of Sindbis virus (SINV) isolates sampled between 1960 and 2014 from countries in the Australasian region and found that G2 viruses were the most frequently and widely sampled, with three distinct sub-lineages defined and no new G6 SINV identified, confirming the geographic restriction of these viruses to south-western Australia [15]. As G2/G3 are collectively a single distinct alphavirus species and G6 is a distinct alphavirus species, it was proposed that they be named Argyle virus and Thomson’s Lake virus, respectively. 

Liu et al. provide insights into the pathogenesis and potential risk of Tembusu virus (TMUV) to mammals [16]. Through their study, they showed that the TMUV HB strain isolated from diseased ducks demonstrated high virulence in BALB/c mice compared with the reference duck TMUV strain and that the 326K at E protein is critical for the mammalian adaption of TMUV. 

Burke et al. define the cynomolgus macaque animal model for aerosolised Venezuelan equine encephalitis virus (VEEV) and demonstrate the importance of challenge dose and viral subtype [17]. They proposed the use of a lower challenge dose (1.0 × 10^3^ PFU) in the aerosol CM model of VEEV disease as NHPs at this dose showed the highest level of viremia, a fever response, and a measurable reduction in complete lymphocyte counts (biomarkers that can demonstrate MCM efficacy).

Wu et al. engineered the ZIKV replicon as mammalian expression vectors and evaluated the potential of ZIKV mini-replicon-based SRIPs as delivery vehicles for heterologous gene expression in vitro and in vivo. This particular study highlights the potential of ZIKV mini-replicon-based SRIPs as promising vehicles for gene delivery applications [18]. 

Crawford et al. performed an RNA-sequencing analysis of the transcriptional response of HEK-293 cells to infection with either mosquito- or mammalian-derived Sindbis virus (SINV), and it was shown that mosquito-derived virus infection leads to a more robust transcriptional response in HEK-293s compared to infection with the BHK-derived virus [19]. They also identified a potential mechanism leading to the more rapid shut-off of host translation and suggested that this mechanism acts to counter the IFN-β-stimulated transcriptional response.

Liang et al. show that hTIM-1 protein can directly interact with Japanese encephalitis virus (JEV) E protein and mediates JEV infection and that this interaction was found to be on specific binding sites between ND114115 in the hTIM-1 IgV domain and K38 of the E protein [20].

Osuna-Ramos et al. investigated the antiviral potential of the cholesterol-lowering drugs atorvastatin and ezetimibe in monotherapy and combination against DENV, ZIKV, and YFV [21]. They demonstrated the potential of atorvastatin and ezetimibe as antiviral agents against flaviviruses in vitro, and their potential was also demonstrated in vivo using AG129 mice infected with DENV 2.

The second section of this Special Issue consists of two review articles by Kuno [22] and Taylor and Rayner [23]. In Kuno’s review article, the author investigates the mechanisms of yellow fever virus (YFV) and the process leading to outbreak occurrence, environmental factors, dispersal, and viral persistence in nature [22]. In Taylor and Rayner’s review article, the authors investigate the immune responses to CHIKV and, in particular, the importance of studying sex as a biological variable by introducing epidemiological studies covering previous CHIKV outbreaks [23]. In their study, it is suggested that while the female sex appears to be a risk factor for chronic CHIKV disease, the male sex is suggested as a risk factor for CHIKV-associated death. 

The third and final section of this Special Issue consists of two brief reports on DENV research by Zuckerman et al. and Wilken et al. [24,25]. Zuckerman et al. performed a phylogenetic analysis of DENV serotypes 1 and 3, which were diagnosed in travelers and Nepalese infected in Kathmandu, Nepal, during the October 2022 outbreak, which suggested that both serotypes may have originated in India [24]. Wilken et al. developed a transiently immunocompromised DENV2 mouse infection model for vaccine testing by treating adult wild-type mice with an IFNAR1-blocking non-cell-depleting antibody (MAR1-5A3) prior to DENV2 infection, and it was shown that these mice seroconverted, with high levels of viral RNA detected in the mice, indicating productive viral replication [25]. 

In summary, the collection of articles included in this Special Issue provides new insights into previously unexplored areas of alphavirus and flavivirus research.
